# Acral melanoma of the heel mimicking a pressure sore

**DOI:** 10.3205/iprs000185

**Published:** 2024-03-04

**Authors:** Matthias Fischer, Anita Sünkenberg, Reem Ali Deeb, Björn Dirk Krapohl

**Affiliations:** 1 Department of Dermatology, Venerology and Allergology, Carl-Thiem-Klinikum Cottbus, Germany; 2 Department of Dermatology and Venerology, Martin-Luther-University Halle-Wittenberg, Halle (Saale), Germany; 3 Department of Dermatology, Helios Klinikum Aue, Germany; 4 Department of Oral and Maxillofacial Surgery, Plastic and Reconstructive Surgery, Carl-Thiem-Klinikum Cottbus, Germany; 5 Berlin Center for Musicians Medicine, Charité – Medical University of Berlin, Germany; 6 Department of Plastic Surgery, Medical University of Lübeck, Germany

**Keywords:** melanoma, pressure ulcers, nursing

## Abstract

**Background:**

The clinical appearance of acral melanoma is diverse and can cause diagnostic difficulties in individual cases.

**Case description:**

We present a clinical case of an 83-year-old patient with a melanoma in the heel area that was initially interpreted as a pressure ulcer, resulting in delayed and more complicated treatment.

**Conclusions:**

Melanomas should be included in the differential diagnosis even in “typical” pressure ulcer areas. Against the background of increasingly poor healthcare in rural areas, an increase in such cases can be expected.

## Zusammenfassung

**Hintergrund:**

Das klinische Erscheinungsbild des akralen Melanoms ist vielfältig und kann in einzelnen Fällen diagnostische Schwierigkeiten verursachen.

**Fallbeschreibung:**

Wir präsentieren einen klinischen Fall einer 83-jährigen Patientin mit einem Melanom im Fersenbereich, das zunächst als Druckgeschwür interpretiert wurde, was zu verzögerter und komplizierterer Behandlung führte.

**Schlussfolgerungen:**

Melanome sollten auch in „typischen“ Druckgeschwürbereichen in die differenzialdiagnostischen Überlegungen einbezogen werden. Vor dem Hintergrund einer zunehmend schlechteren Gesundheitsversorgung in ländlichen Gebieten ist mit einer Zunahme solcher Fälle zu rechnen.

## Introduction

Malignant melanoma is the most fatal skin disease [1]. Malignant melanomas in the foot and ankle region account for 3–15% of all melanomas [2]. Acral melanomas have a worse prognosis compared to melanomas in other locations [3]. According to current research, even in non-metastatic stages (I–II), local recurrences occur in 27.9% of cases [4]. Complicating matters is the fact that acral localized melanomas often present with an atypical clinical appearance, leading to delays in further diagnosis and treatment. Acral melanomas have been described in the literature that have occurred under the image of plantar warts, various benign tumors, hemorrhages, infections, and nonspecific ulcerations [5]. Thus, acral melanomas can, albeit rarely, present with the appearance of a pressure sore (decubitus) and must therefore be considered in the differential diagnosis of pressure ulcers. Pressure ulcers are a common phenomenon in nursing. The prevalence in long-term care is between 2–5% and 2–4% in patients treated in hospital [6]. The diagnosis is usually made clinically and is based on the distribution (pressure-loaded body areas) and the clinical morphology depending on the degree of the pressure ulcer. Arterial, rarely mixed arterial-venous circulatory disorders and vasculitis should be considered in the differential diagnosis. 

Below, we report of a case of a melanoma located on the heel, which was initially misinterpreted as a pressure ulcer.

## Case description

**Figure 1:**
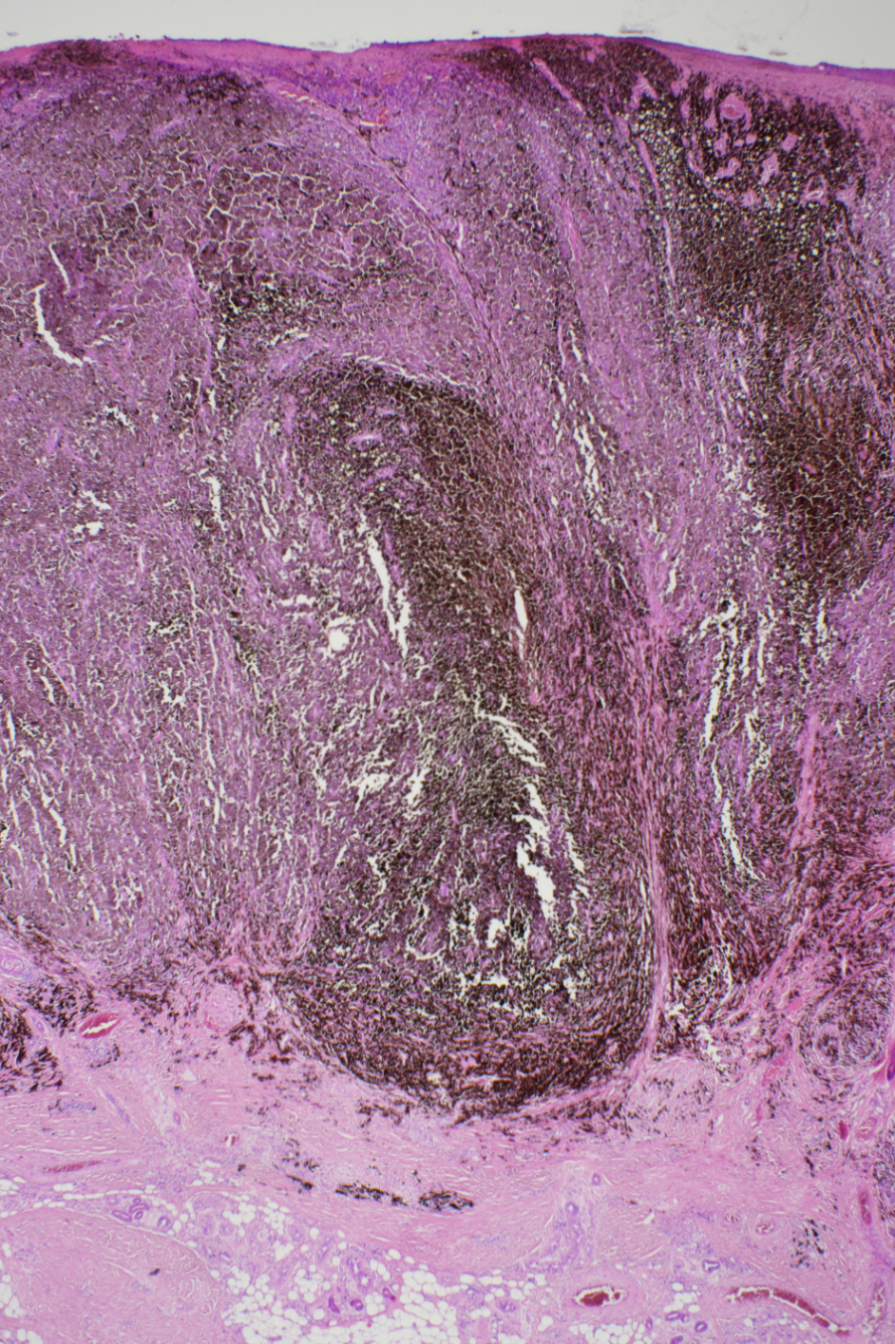
Histology of the excised melanoma (haematoxylin and eosin stain)

**Figure 2:**
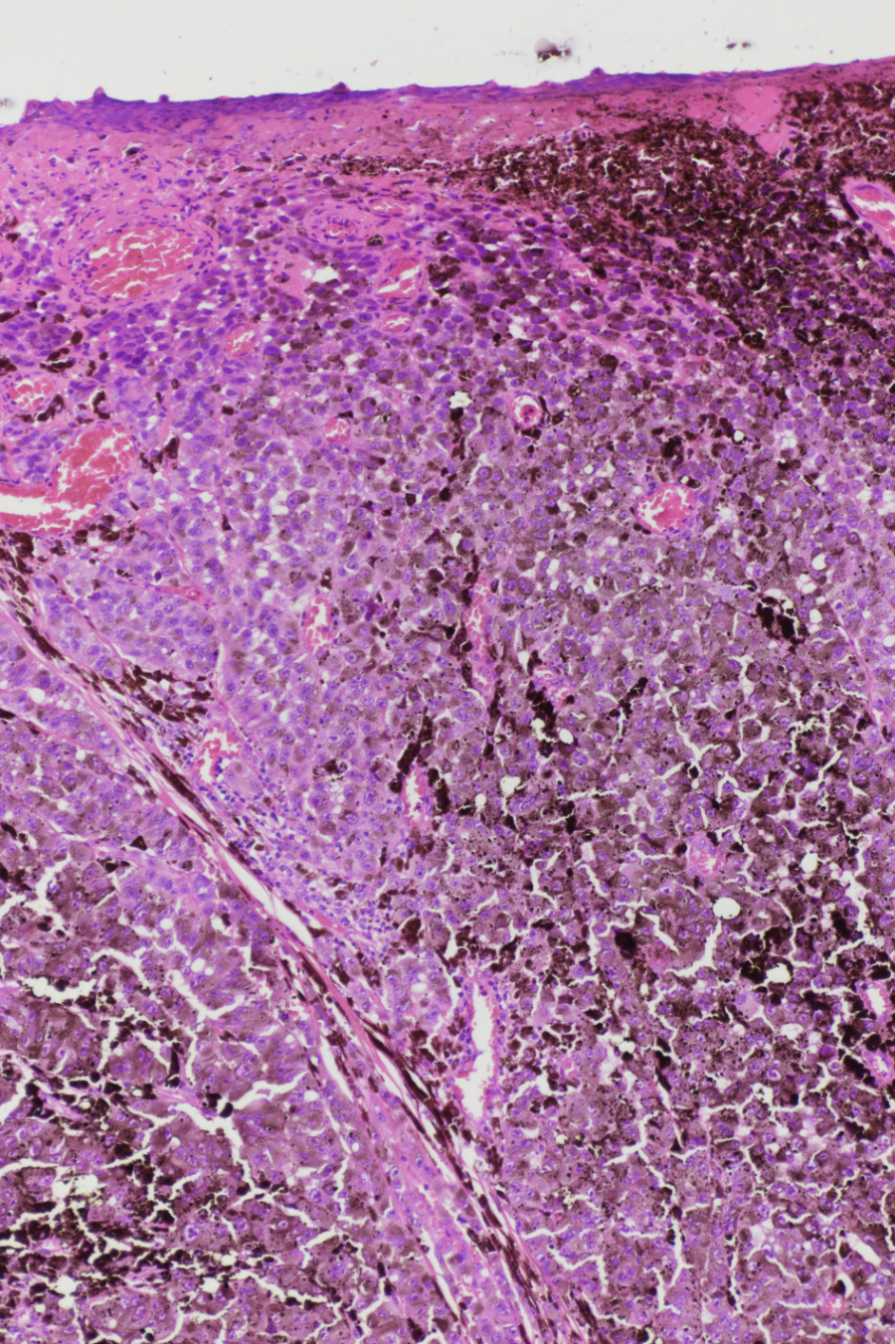
Histology of the excised melanoma (haematoxylin and eosin stain, higher magnification): Typical asymmetrical and poorly circumscribed lesions with architectural disturbance and marked cytological atypia, nests of melanocytes with variable size, increased number of cell apoptosis

**Figure 3:**
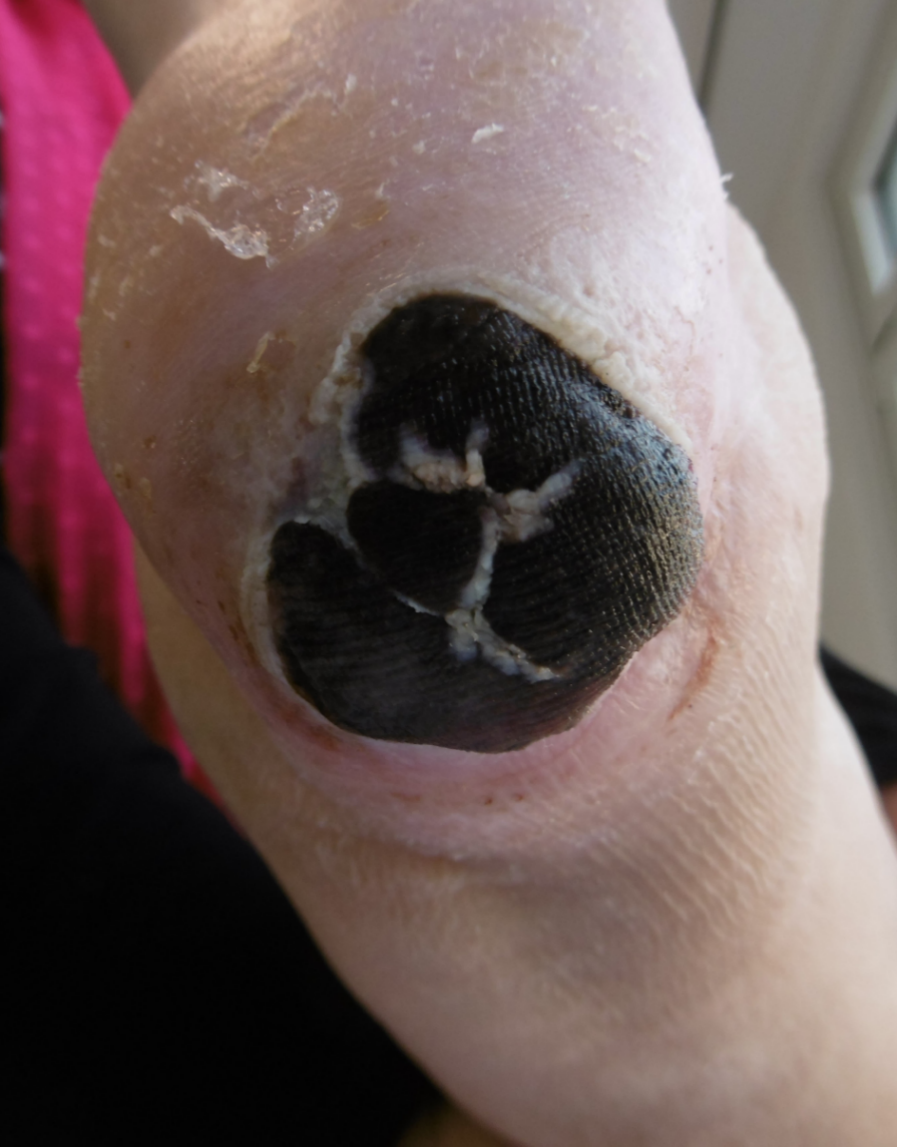
Melanoma of the heel, initially misdiagnosed as a pressure sore

**Figure 4:**
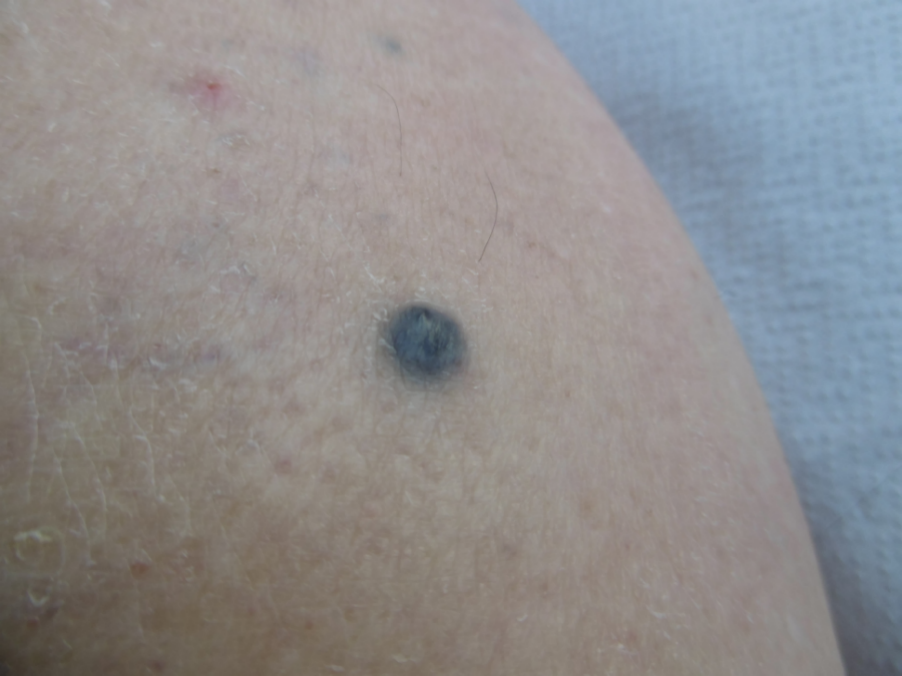
Cutaneous in-transit metastasis of the ipsilateral lower leg

An 83-year-old Caucasian woman noticed an erythema in the area of the left heel at least four months ago, which subsequently turned black. A pressure-relieving treatment carried out under the clinical suspicion of a pressure ulcer, as well as various wound dressings, were unable to stop further progression. A biopsy then showed a melanoma (Figure 1 (Fig. 1) and Figure 2 (Fig. 2)). Upon admission to the hospital, a 4 cm black, bleeding, sharply demarcated tumor was found, which was cap-like and located on the heel (Figure 3 (Fig. 3)). Additionally, on the left lower leg, there was an 8 mm-sized, black-gray nodule, clinically corresponding to an in-transit metastasis (Figure 4 (Fig. 4)).

The tumor on the heel and the in-transit metastasis were excised. The defect on the heel was treated with vacuum therapy. Histologically, a completely resected nodular malignant melanoma with a maximum tumor thickness of 6.2 mm was found on the heel. The suspicion of an in-transit metastasis was confirmed in the tumor on the lower leg, which was also completely excised. Staging revealed left inguinal lymph node metastases and possibly left iliac lymph node metastases in the CT scan. The tumor stage classification according to the TNM classification was pT4b, cN3, cM0, L1, V1, Pn0. The clinical stage according to the AJCC classification was IIIC. The melanoma tumor marker S100 in the serum was elevated at 0.32 µg/l (normal 58 range: <0.15 µg/l). The lactate dehydrogenase (LDH) as a nonspecific measure of tumorburden was within normal range. Molecular pathological examinations carried out to prepare for possible systemic drug therapy showed a wild type for BRAF and N-RAS, so the use of tyrosine kinase inhibitors was not appropriate. Due to multiple comorbidities (heart failure, kidney failure), further surgical intervention was waived after consultations in the interdisciplinary tumor board. Instead, drug treatment with a checkpoint inhibitor (nivolumab) was initiated with a palliative approach, leading to stable disease.

## Discussion

Malignant melanoma is a malignant tumor originating from pigment cells with primary lymphogenic metastasis. Various subtypes of malignant melanoma are described, which were reclassified by the World Health Organization (WHO) in 2018 (Table 1 (Tab. 1)) [7]. The previous classification based solely on descriptive clinical and histological aspects has been abandoned in favor of additional consideration of molecular pathological findings [1]. Mutations of the BRAF gene, which has a significant impact on tumor growth control, are at the center of attention. The discovery of cellular mechanisms that influence the growth of malignant melanoma has led to the development of new drugs that enable individualized and targeted therapy. The groups of tyrosine kinase inhibitors (a prerequisite: BRAF gene mutation) and checkpoint inhibitors are the focus of attention. Checkpoint inhibitors deactivate mechanisms that tumor cells use to evade the body’s immune defenses. Despite the significant advances in the pharmacological treatment of melanoma, surgical excision of tumors remains the first treatment option that often suffices to control tumor progression. In the case of acral melanomas, specific mutations (CRKL and GAB2) have been identified, which can explain a repeatedly described poor response to immunotherapy in the literature [3]. Therefore, early resection of malignant melanoma, and thus lower tumor thickness, is still the decisive factor in the prognosis of affected patients with acral melanomas. Sondermann et al. were able to show in a retrospective analysis that 30% of melanomas on the feet were unfortunately not detected at the initial medical examination [8]. The case presented here serves as a good example for this. The mistaken initial assessment of the acral melanoma as a pressure sore can be explained by the fact that the clinical appearance of a sharply demarcated, heel-located, extensive black tumor (Figure 3 (Fig. 3)) exhibited aspects of necrosis, which are typical of a pressure sore. This is supported by information in the literature, where, in the presence of corresponding pressure sore risk factors, the malignant melanoma was initially misinterpreted as damage from pressure [8]. Risk factors for misdiagnosis include concurrent diabetes with diabetic foot syndrome and advanced age [9]. Additionally, it must be considered that acral melanomas can also appear as non-pigmented tumors (amelanotic melanomas) [10]. Recent findings suggest that mechanical stress plays no significant role in the development of melanoma, given the identification of specific mutations in acral melanomas [3], and only isolated cases of melanomas occurring in long-existing pressure sores at other locations are found in the literature [11].

Primary care in the medical sector plays a crucial role in early detecting of lesions suspicious for a melanoma. However, early detection of melanoma is challenging in individual cases, particularly concerning clinically atypical melanoma or melanoma in unusual or difficult-to-observe anatomical regions. In addition, malignant melanomas in body regions with little exposure to sunlight are not included in the differential diagnosis. Therefore, close interdisciplinary collaboration between nursing staff and physicians is absolutely necessary to identify tumor-suspected lesions and to treat them promptly.

## Conclusion

In our case, it is assumed that the melanoma on the heel developed randomly. However, this case also demonstrates that insufficient critical assessment of the findings can lead to misdiagnosis, resulting in delays in diagnosis and treatment and a poorer prognosis. This is particularly true for patients with tumors in “typical” decubitus locations and concomitant risk factors for pressure or ischemia-related ulcers. The risk could even increase in the future if the decline in specialist dermatological care continues. The often difficult staffing situation in outpatient care and nursing homes exacerbates the situation further. Expanding teledermatological services may be one approach to reduce the risk of misjudging acral melanomas. In cases of doubt, especially when there is rapid growth of the lesion and bleeding, an early biopsy is helpful.

Regardless of technological advances, early detection of melanoma continues to play a central role, in which various nursing facilities have an important and responsible function.

## Notes

### Competing interests

The authors declare that they have no competing interests.

## Figures and Tables

**Table 1:**
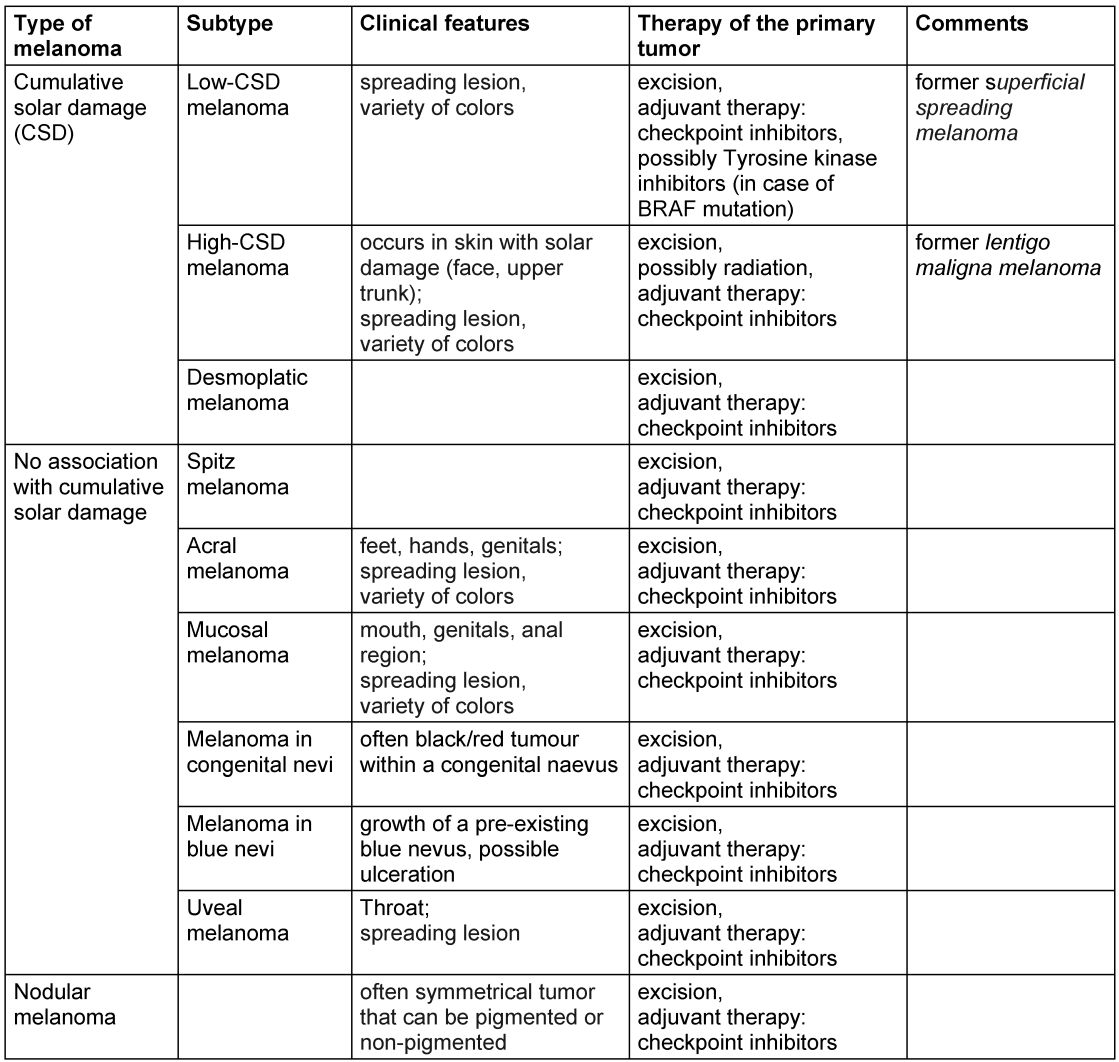
Summary of the malignant melanomas of the skin (WHO melanoma classification)
